# Recognizing Banknote Fitness with a Visible Light One Dimensional Line Image Sensor

**DOI:** 10.3390/s150921016

**Published:** 2015-08-27

**Authors:** Tuyen Danh Pham, Young Ho Park, Seung Yong Kwon, Dat Tien Nguyen, Husan Vokhidov, Kang Ryoung Park, Dae Sik Jeong, Sungsoo Yoon

**Affiliations:** 1 Division of Electronics and Electrical Engineering, Dongguk University, 30, Pildong-ro 1-gil, Jung-gu, Seoul 100-715, Korea; E-Mails: phamdanhtuyen@gmail.com (T.D.P.); fdsarew@hanafos.com (Y.H.P.); sbaru07@dgu.edu (S.Y.K.); nguyentiendat@dongguk.edu (D.T.N.); vokhidovhusan@nate.com (H.V.); jungsoft97@dongguk.edu (D.S.J.); 2 Kisan Electronics, Sungsoo 2-ga 3-dong, Sungdong-gu, Seoul 133-831, Korea; E-Mail: ssyoon@kisane.com

**Keywords:** classification of banknote fitness, one-dimensional line image sensor of visible light, discrete wavelet transform, linear regression analysis, support vector machine

## Abstract

In general, dirty banknotes that have creases or soiled surfaces should be replaced by new banknotes, whereas clean banknotes should be recirculated. Therefore, the accurate classification of banknote fitness when sorting paper currency is an important and challenging task. Most previous research has focused on sensors that used visible, infrared, and ultraviolet light. Furthermore, there was little previous research on the fitness classification for Indian paper currency. Therefore, we propose a new method for classifying the fitness of Indian banknotes, with a one-dimensional line image sensor that uses only visible light. The fitness of banknotes is usually determined by various factors such as soiling, creases, and tears, *etc*. although we just consider banknote soiling in our research. This research is novel in the following four ways: first, there has been little research conducted on fitness classification for the Indian Rupee using visible-light images. Second, the classification is conducted based on the features extracted from the regions of interest (ROIs), which contain little texture. Third, 1-level discrete wavelet transformation (DWT) is used to extract the features for discriminating between fit and unfit banknotes. Fourth, the optimal DWT features that represent the fitness and unfitness of banknotes are selected based on linear regression analysis with ground-truth data measured by densitometer. In addition, the selected features are used as the inputs to a support vector machine (SVM) for the final classification of banknote fitness. Experimental results showed that our method outperforms other methods.

## 1. Introduction

In recent years, automatic payment facilities such as vending machines and automatic teller machines (ATMs) have become more and more popular. As a result, the importance of correctly recognizing and classifying banknotes has increased. This problem consists of not only automatically sorting banknotes by denominations, sides, and directions, but also in determining the fitness of those banknotes. By fitness, we mean determining which banknotes are suitable for recirculation and which should be replaced by new ones. If a fit banknote is recirculated frequently, the cost for printing that banknote can be greatly reduced [1]. In addition, if an unfit banknote is replaced with a new one, the processing speed and accuracy of banknote dispensing in ATMs can be greatly enhanced.

There has been previous research on the classification of banknote fitness with regard to various paper currencies. Based on research on the efficient use of banknotes by the Dutch Central Bank [1], soiling is one of the main characteristics that degrade the fitness of banknotes for circulation [1,2]. This characteristic was taken into account in several studies [1,3,4,5], using color images of banknotes. Geusebroek *et al.* [1] and Balke *et al.* [5] proposed a machine learning method that classified Euro banknotes by the mean and standard deviation of intensity values extracted from overlapping rectangular regions in the channels of banknote images. These values included intensity, color (RGB), and color combinations of YB and RG channels. The classifier used in [1] and [5] was a combination of simple linear weak classifiers using the AdaBoost algorithm. Aoba *et al.* [6] proposed an approach for classifying Euro banknotes that used visible and infrared (IR) images as input data. The system in [6] is composed of a classification part that uses a three-layered perceptron, and a validation part that uses a radial basis function (RBF) network for rejecting unfit data. A neural network was also used to classify Chinese banknotes (RMB) [7]. In this approach, the gray-level histogram of a banknote image was used as the feature vector for the neural network using a sine basis function. In [8], they newly proposed the method of recognizing Bangladeshi banknote by using web-camera for visually impaired people. With the Bangladeshi banknotes of white paper background, their system shows a recognition accuracy of 89.4%, and that of 78.4% with banknotes with complex backgrounds.

There was also research conducted on the classification of Indian banknotes (Rupees), but these approaches focused on the classification of the denomination (type of banknote) [9,10,11]. An embedded-system approach for Indian currency recognition was proposed by Pathrabe *et al.* [12]. This approach focused on counterfeit banknote detection using features extracted from the HSV color space and a neural network classifier. To recognize fake Indian banknotes [13], charge-coupled device (CCD) cameras with visible, ultraviolet (UV), and IR lights were used to detect the security features on banknotes. These security features included watermarks and latent images, which help to detect counterfeit currency.

Although research has been carried out on the automatic classification of Indian currency notes, little of this research focused on classifying the fitness of banknotes. In addition, most of the previous works used multiple sensors. Using multiple sensors can make it easier in classification task by increasing the number of discriminating features. However, it leads to complexity in hardware implementation, and increase of processing time with multiple images by multiple sensors.

To overcome these problems, we proposed a method based on a discrete wavelet transform (DWT) of grayscale Rupee banknote images captured only by a visible light sensor. The fitness of banknotes is usually determined by various factors such as soiling, creases, and tears, *etc*., but we just consider the soiling of the banknote in our research. The fitness of the banknote is classified based on the features extracted from regions of interest (ROIs) that contain little texture. The 1-level DWT extracts the features for discriminating fit and unfit banknote classes. The optimal DWT features that best represent the fitness and unfitness of banknotes are selected based on linear regression analysis with ground-truth data. In addition, the selected features are used as the inputs to the support vector machine (SVM) for the final classification of banknote fitness. Table 1 compares previous research related to banknote fitness classification, as well as our proposed method.

**Table 1:** Comparison of previous work and the proposed method.

Category		Method	Advantages	Disadvantage
Multiple sensor-based method		-Evaluating the five soiling levels of Euro banknotes by using various sensors [4].	Using information by various sensors allows for the extraction of more discriminating features	-Focuses on analyzing the soiling property of the banknotes without proposing a solution for automatically classifying the fitness of banknotes [4].
		-Denomination classification using visible and IR sensors [6].		-Mainly focuses on denomination classification [6] and counterfeit banknote detection [13].
		-Detecting fake banknotes by CCD cameras with visible, UV, and IR lights [13].		-Using multiple sensors leads to complexity in hardware implementation and an increase in processing time with multiple images from multiple sensors.
Single sensor-based method	Color sensor-based method	-Features are extracted from banknote images of various color channels [1,5]. -Detecting counterfeit Indian banknotes using features from HSV color space and neural network classifier [12].	Using a single sensor causes simplicity in the algorithm and system implementation with reduced processing time	-Banknote images with multiple color channels must be acquired, and a large number of features based on many weak classifiers must be combined, thus reducing the processing speed [1,5]. -Mainly focuses on counterfeit banknote classification [12].
	Gray sensor-based method	Chinese banknote classification using neural network based on the features of gray-level histogram [7] -Fitness classification based on DWT and SVM (**proposed method**)	-Fast image acquisition by single gray sensor with less memory usage	-Mainly focuses on banknote classification [7]. -Additional procedure for SVM training is required (**proposed method**)

This paper is organized as follows: Section 2 describes the proposed method in detail. The experimental results are given in Section 3. Finally, conclusions and future works are explored in Section 4.

## 2. Proposed Method

### 2.1. Overview of the Proposed Method

**Figure 1:**
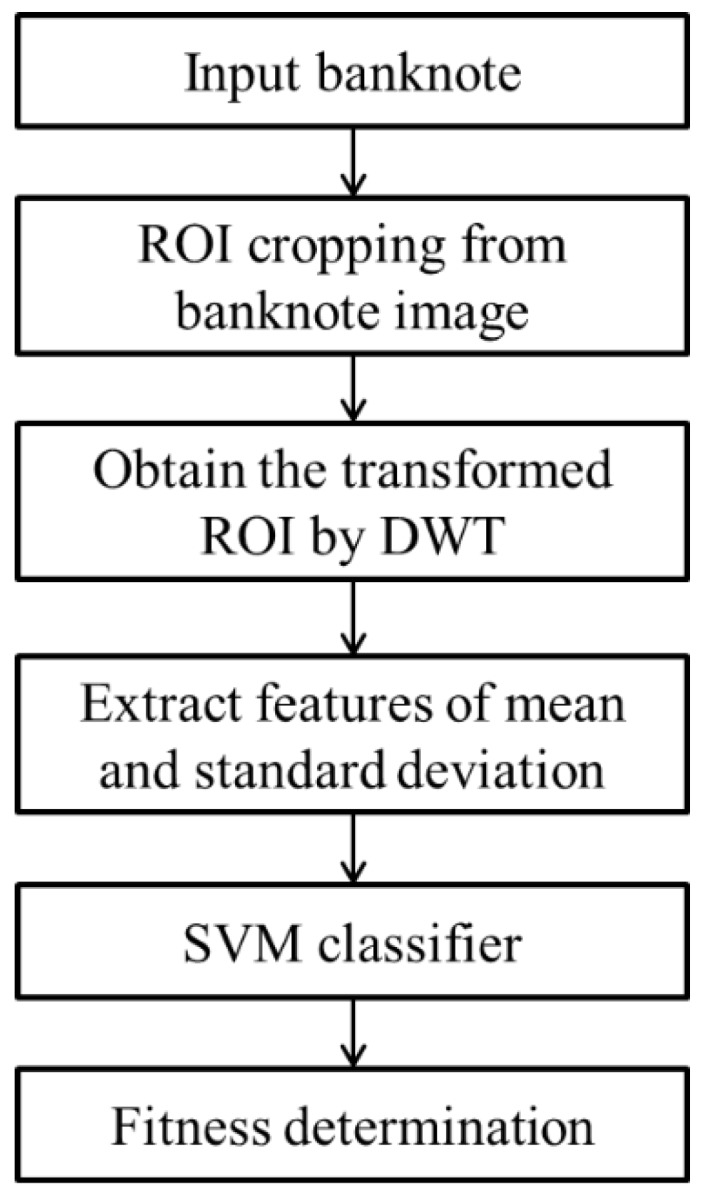
Flowchart of the proposed method.

Figure 1 shows the overview of the proposed classification method. After acquiring the input banknote images, the ROIs are cropped and decomposed by DWT. The 1-level DWT decomposition produces a two-dimensional signal that consists of four sub-bands: LL, LH, HL, and HH. Detailed explanations are shown in the following sections. From each sub-band, the mean and standard variation values are calculated; thus, eight features are extracted by using DWT from the ROI for each input image. Among the eight features, an optimal two features are selected based on linear regression analysis. The two features are fed into an SVM classifier. In the last step, the input banknote is determined to be fit or unfit for recirculation based on the output of the SVM.

### 2.2. ROI Cropping and Feature Extraction

**Figure 2:**
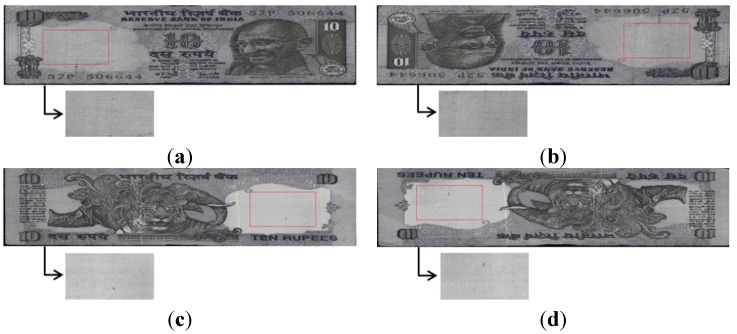
Example of input images in 4 directions and corresponding ROIs of a banknote: (**a**) A direction; (**b**) B direction; (**c**) C direction; (**d**) D direction.

We acquired the banknote image by a commercial banknote-counting machine. Because the banknote-counting machine has limitations of size and cost, it captures the banknote image of visible light by a one-dimensional (line) sensor that differs from the conventional camera using a two-dimensional (area) sensor. Therefore, at each triggering time, a space (row by column) image is not captured, but one line (row) image is acquired. While the input banknote is moving through the roller device within the banknote-counting machine at fast speed (higher than 1000 ppm (pulses per minute)), the line image is captured with a visible light emitting diode (LED). The number of pixels of one line image is 1584, and a total of 464 line images are obtained by this system. Therefore, by concatenating the 464 line images sequentially, a two-dimensional banknote image of 1584 × 464 pixels is finally acquired, and the area of banknote is located by a commercial corner detection algorithm which is already built in to the banknote-counting machine used in our experiment. Due to the detection of the area of banknote (excluding the background) in the captured image, the problems of displacement and rotation of banknote area can be solved in our research, and we can obtain the correct area of banknote as shown in Figure 2.

The input banknotes are usually captured in four cases in terms of side and direction: forward and reverse images of the front side, and forward and reverse images of the back side. These are denoted by A–D directions in our research, respectively, as shown in Figure 2. Because both the front and backsides of Indian banknotes have areas that do not include visible texture, these are considered as the ROIs in our proposed method. We chose the ROIs where the amount of banknote type information (patterns, figures, symbols, and numbers, *etc*.) is minimal. That is because the mean and standard deviation of the region (where the banknote type information are included) are affected by the amount of the banknote type information even with the banknote images of same soiling level, which makes it difficult to correctly discriminate the fit and unfit banknotes. Figure 2 shows examples of the Indian Rupee in the four directions, and the ROIs cropped from these banknote. The banknote-counting machine (used in our experiment) already has the functionality of automatically recognizing the A–D directions with the kinds of banknote, and this functionality was implemented as the commercial software. We manually defined the positions of ROIs according to the directions (A–D) and the kinds of banknote. Based on this information (the positions of ROIs) and the recognition results of the directions and kinds of banknote by the banknote-counting machine, the ROIs for extracting features are automatically detected in our research as shown in Figure 2.

Using the cropped ROIs, features are extracted by DWT. Previous research used DWT to obtain features from banknote images, but mostly focused on classifying types of banknotes [14,15]. DWT has been widely used for extracting the texture feature in the domain of image signal processing [16,17]. In this research, in order to obtain the features for discriminating fitness, we apply DWT on the ROIs cropped from the banknote image. The ROI images are normalized by resizing them to 256 × 256 pixels before the DWT. The 1-level DWT decomposition produces a two-dimensional signal that consists of four subbands: LL, LH, HL, and HH. LL denotes the area of low-frequency components in both the horizontal and vertical directions. LH and HL are the areas where low frequency components exist in one of the horizontal and vertical directions, and high frequency components exist in the other direction. HH represents the area of high-frequency components in both the horizontal and vertical directions [16]. In our research, the DWT was performed by Haar and Daubechies functions [17], and their performances were compared. In addition, we performed the experiments of comparing the accuracies of discriminating the fit and unfit banknotes according to the various levels of DWT. Experimental results showed that the 1-level DWT outperforms the other levels DWT, and we used the 1-level DWT.

**Figure 3:**
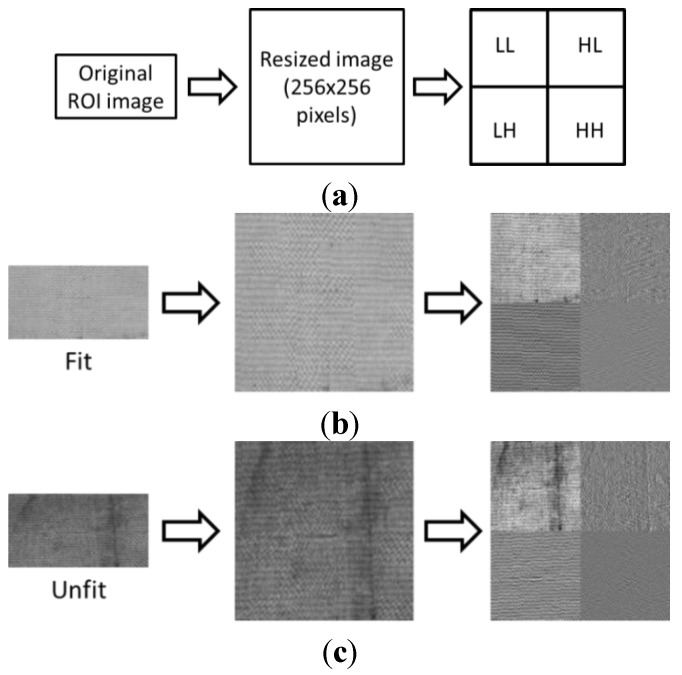
DWT with fit and unfit banknotes: (**a**) procedure of DWT and resulting images of DWT with (**b**) fit banknote and (**c**) unfit banknote.

Figure 3 shows examples of Haar wavelet transforms for ROIs for fit and unfit banknotes after they were resized to 256 × 256 pixels using bilinear interpolation. In detail, the left images of Figure 3b,c show the original ones of ROI extracted from input banknote. The middle ones are the size normalized images of 256 × 256 pixels which are used for the transform of DWT. The right images are the ones obtained by 1-level DWT. For each sub-band, we calculated the mean (μ) and standard deviation (σ) values. Thus, by using 1-level DWT, we obtain eight feature values from four sub-bands. This feature vector can be written as (μ*_LL_*, σ*_LL_*, μ*_HL_*, σ*_HL_*, μ*_LH_*, σ*_LH_*, μ*_HH_*, σ*_HH_*). As shown in Figure 3b,c, the fit image has the characteristics of being brighter and having less texture than the unfit image, whereas the unfit image usually includes more soiling on its surface. Therefore, the mean of the fit image is usually higher than that of the unfit image, whereas the standard deviation of the fit image is lower than that of the unfit image because we just consider the soiling level of the banknote as the measure of fitness in our research.

### 2.3. Selection of Optimal Features Using Regression Analysis

Given the eight feature values (μ*_LL_*, σ*_LL_*, μ*_HL_*, σ*_HL_*, μ*_LH_*, σ*_LH_*, μ*_HH_*, σ*_HH_*), optimal values are selected based on statistical analysis (linear regression analysis). Regression analysis is a statistical method for estimating the relationships between two variables. In this research, we use linear regression, in which the relationships between a scalar dependent variable *y* (reference data) and explanatory variables *x* (input data) are modeled by linear predictor functions *r*(*x*). A regression line is determined to minimize the distance between the measured and predicted values of *y* [18]. The quality of fitting in the model is evaluated by the coefficient of determination *R^2^*, which indicates how well data points fit the regression line. *R^2^* is calculated by the following Equation [19]:

(1) $$R^{2}=1-\frac{\sum_{i=1}^{n}{\left[y_{i}-r(x_{i})\right]}^{2}}{\sum_{i=1}^{n}{\left(y_{i}-\bar{y}\right)}^{2}}$$

where $\bar{y}$ is the mean of the reference data. *R^2^* receives the values in a range of 0 to 1, in which 1 indicates the cases where the regression line perfectly fits the data, and 0 implies that there is no linear relation between the two variables.

**Figure 4:**
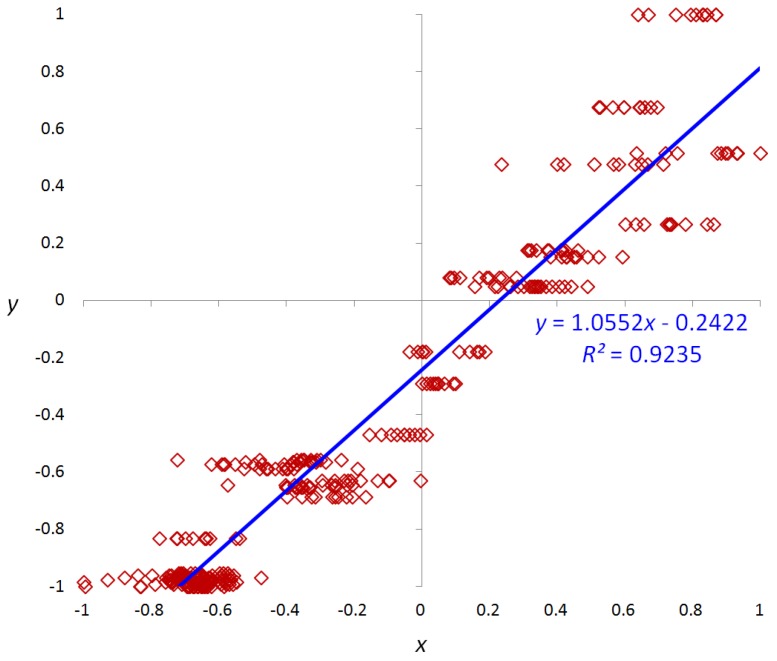
Example of linear regression analysis on two variables of *x* and *y*.

In our method, we use one of the eight feature values (μ*_LL_*, σ*_LL_*, μ*_HL_*, σ*_HL_*, μ*_LH_*, σ*_LH_*, μ*_HH_*, σ*_HH_*) as the *x* (input data) of the regression method. As the *y* (reference data) of the regression method, we use the measured value by densitometer [20] based on a soiling level defined by the State Bank of India [21]. By using densitometer, we can measure the ground-truth value of reflectance on the surface of banknote. In general, the banknote of fitness usually shows the higher value of reflectance than that of unfitness. Therefore, we used the reflectance on the surface of banknote by densitometer as the ground-truth value for selecting the optimal features. Based on linear regression results, the two features having the highest *R^2^* values are chosen as the two features for discriminating the fitness and unfitness of the banknote. These features are used as the two inputs to the SVM classifier. Figure 4 shows an example of linear regression analysis on two variables *x* and *y*, scaled to a range of −1 to 1 in the case of using σ*_LH_* feature extracted by Daubechies DWT from C direction images of 50 Rupee.

### 2.4. SVM Training and Testing

In the last step, fit and unfit banknotes are classified by using SVM with the two input features selected by the regression method. The SVM has been widely used as supervised classifier in the domain of pattern recognition [17,22]. The discriminant function of SVM is given as follows [17]:

(2) $$f(x)=\mathrm{sgn}\left(\sum_{i=1}^{N}y_{i}\alpha_{i}K(x_{i},x)+\text{β}\right)$$

where *N* is the number of data points, *x_i_* represents training points, and *y_i_* are the indicator vectors representing the class labels such as $y_{i}\in \left\{-1,1\right\}$, in which –1 stands for “class 1” and 1 stands for “class 2.” In our research, we define “class 1” and “class 2” as the classes of fit and unfit banknotes, respectively. *K*(*x,y*) is the kernel function. We compared the accuracy of the following four kernel functions, which are popularly used in SVM classifiers [17,22]:

(3) $$\begin{array}{ll} Radial\;basis\;function\;\left(RBF\right)\;kernel: & K(x,y)=\exp(-\text{γ}{\left\|x-y\right\|}^{2}),\;\text{γ}>0\\ Linear\;kernel: & K(x,y)=x^{T}y\\ Polynomial\;kernel: & K(x,y)={\left(\text{γ}x^{T}y+r\right)}^{d},\;\text{γ}>0\\ Sigmoid\;kernel: & K(x,y)=\tanh(\text{γ}x^{T}y+r) \end{array}$$

where γ, *r*, and *d* are the parameters of the kernels. The optimal kernel is selected by a training process in terms of the minimum classification error (equal error rate–EER). The EER is the error rate when the difference between the types 1 and 2 error rates is smallest [17]. In the testing phase, the types 1 and 2 error rates and EER are calculated to evaluate the accuracy of the proposed method. A type 1 error indicates that we have misclassified fit banknotes into unfit ones, whereas type 2 errors indicate the opposite.

## 3. Experimental Results 

We used the Indian banknote database for our experiments. This database is composed of banknotes in denominations of 10, 20, 50, 100, and 500 Rupees. Each banknote was captured in four directions, as shown in Figure 2. The experiments on fitness classification were conducted separately on the banknote images of four directions from the five denominations. The numbers of banknote images in our experimental database are shown in Table 2. In our experiments, we performed two-fold cross-validation. That is, each data set of banknote images was randomly divided into two parts in the first trial. One of the parts was used for training, and the other was used for testing. In the second trial, the data sets for training and testing were swapped and the experiments were performed again. From those results, we calculated the average of the two accuracies by two trials.

**Table 2:** Number of images in Indian banknote database.

Denominations	A Direction	B Direction	C Direction	D Direction
10 Rupee	1040	1020	1020	1020
20 Rupee	680	670	710	710
50 Rupee	620	620	650	650
100 Rupee	1540	1550	1520	1530
500 Rupee	930	910	950	960

**Table 3:** Experimental results of DWT features selection based on linear regression. (*Denom*. and *Dir*. are denominations and directions, respectively. *Std* indicates standard deviation.)

Denom.	Dir.	Haar DWT				Daubechies DWT			
		Train 1—Test 2		Train 2—Test 1		Train 1—Test 2		Train 2—Test 1	
		Selected Features	*R^2^*	Selected Features	*R^2^*	Selected Features	*R^2^*	Selected Features	*R^2^*
10 Rupee	A	LL mean	0.6909	LL mean	0.8266	LL mean	0.6833	LL mean	0.8327
		LL std	0.6437	LL std	0.7720	LL std	0.6365	LL std	0.7792
	B	LL mean	0.6654	LL mean	0.8443	LL mean	0.6812	LL mean	0.8284
		LL std	0.6099	LL std	0.7455	LL std	0.6109	LL std	0.7477
	C	LH std	0.9026	LH std	0.9296	LH std	0.8888	LH std	0.9197
		LL mean	0.8055	HH std	0.8691	LL mean	0.8176	HL std	0.8692
	D	LH std	0.9052	LH std	0.9274	LH std	0.8845	LH std	0.9164
		LL mean	0.8300	HH std	0.8628	LL mean	0.8394	HL std	0.8587
20 Rupee	A	LL std	0.7222	LL std	0.8243	LL std	0.7244	LL std	0.8238
		LL mean	0.5351	LL mean	0.6733	LL mean	0.5682	LL mean	0.6864
	B	LL std	0.7000	LL std	0.8075	LL std	0.6917	LL std	0.8239
		LL mean	0.5791	LL mean	0.6760	LL mean	0.5799	LL mean	0.6746
	C	LH std	0.8287	HL std	0.7775	LH std	0.8034	HL std	0.7834
		HL std	0.7781	LH std	0.7412	HL std	0.7783	LH std	0.7439
	D	LH std	0.8514	LH std	0.7314	LH std	0.8282	LH std	0.7526
		HL std	0.7964	LL mean	0.7096	HL std	0.7962	LL mean	0.7105
50 Rupee	A	LL std	0.9018	LL std	0.9249	LL std	0.9043	LL std	0.9224
		LH std	0.8949	LL mean	0.8764	LL mean	0.8526	LL mean	0.8887
	B	LL std	0.8934	LL std	0.9315	LL std	0.8960	LL std	0.9274
		LH std	0.8778	LL mean	0.8762	LL mean	0.8557	LL mean	0.8817
	C	LH std	0.9611	LH std	0.9390	LH std	0.9511	LH std	0.9235
		LL mean	0.9558	HL std	0.9144	LL mean	0.9471	LL mean	0.9087
	D	LH std	0.9627	LH std	0.9450	LH std	0.9518	LL mean	0.9414
		HL std	0.9489	LL mean	0.9439	LL mean	0.9418	LH std	0.9374
100 Rupee	A	LH std	0.8213	LH std	0.8307	LH std	0.7635	LL std	0.8234
		LL mean	0.7222	LL std	0.8146	LL mean	0.7210	LH std	0.7917
	B	LH std	0.8170	LH std	0.8249	LL mean	0.7160	LL std	0.8313
		LL mean	0.7395	LL mean	0.8062	LL std	0.7141	LL mean	0.8007
	C	LH std	0.8599	LH std	0.8817	LH std	0.8276	HL std	0.8723
		HL std	0.8171	HL std	0.8638	HL std	0.7986	LH std	0.8694
	D	LH std	0.8502	LH std	0.9030	LH std	0.8112	LH std	0.8476
		HL std	0.8073	HL std	0.8858	LL mean	0.7883	HL std	0.8448
500 Rupee	A	LL std	0.6582	LL std	0.5521	LL std	0.6448	LL std	0.5581
		HL mean	0.4041	HL mean	0.3839	HL mean	0.3582	LL mean	0.3580
	B	LL std	0.4907	LL std	0.5184	LL std	0.5108	LL std	0.5510
		HH std	0.2833	LL mean	0.3015	HL std	0.2627	LL mean	0.3174
	C	LH std	0.9314	LH std	0.8388	LH std	0.9105	LH std	0.7695
		HL std	0.9309	HL std	0.7307	HL std	0.8899	LL mean	0.6551
	D	HL std	0.9291	LH std	0.8523	LH std	0.9270	LH std	0.8016
		LH std	0.9203	HL std	0.7639	LL mean	0.8959	LL mean	0.6472

In our experiments for feature selection, the two optimal features which showed the best-fitting result based on linear regression analysis (as explained in Section 2.3) were selected among the eight feature values (μ*_LL_*, σ*_LL_*, μ*_HL_*, σ*_HL_*, μ*_LH_*, σ*_LH_*, μ*_HH_*, σ*_HH_*) that were obtained by DWT. The feature selection results for the Haar and Daubechies DWT of banknote images are shown in Table 3.

We divided the database into two parts, (Parts 1 and 2) for training and testing. Thus, in Table 3, “Train 1—Test 2” means that Part 1 was used for training, and Part 2 for testing. In addition, “Train 2—Test 1” means that Part 2 was used for training, and Part 1 was used for testing. In each case, *R^2^* values are calculated by using the training data. As shown in Table 3, we selected the two features that had the highest *R^2^* values in Equation (1) according to different directions of A–D. After obtaining the optimal discriminant features, we determined the fitness of the banknote by using the SVM classifier. For SVM training and testing, we used the two parts of the database from the previous experiments in the feature-selection step. Since the training data was normalized by min-max scaling to the range of 0 to 1, the testing data set was also normalized based on the min-max range of training data.

**Figure 5:**
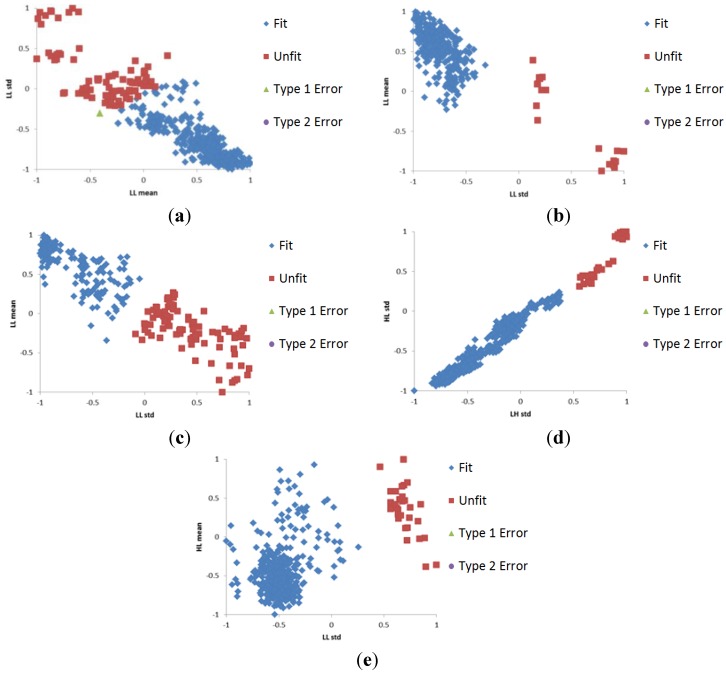
Examples of data distributions of training datasets in SVM classifications by DWT with (**a**) Daubechies kernel on 10 Rupees in the A-direction; (**b**) Haar kernel on 20 Rupees in the B-direction; (**c**) Daubechies kernel on 50 Rupees in the B-direction; (**d**) Haar kernel on 100 Rupees in the C-direction; and (**e**) Haar kernel on 500 Rupees in the A-direction.

The optimal SVM parameters and kernel were obtained by the training process. We conducted experiments using SVM with four kernels of RBF, linear, polynomial, and sigmoid of Equation (3), using the LIBSVM tool [23]. Figure 5 shows examples of distributions of training data in the feature space.

Using the trained SVM, the results obtained by Haar DWT with testing data are shown in Table 4. Results obtained by Daubechies DWT with testing data are shown in Table 5. As previously explained, we performed two-fold cross-validation and obtained the average EER. As explained in Section 2.4, the EER is the error rate when the difference between the types 1 and 2 error rates is smallest [17]. In the testing phase, the types 1 and 2 error rates and EER are calculated to evaluate the accuracy of the proposed method. A type 1 error indicates that we have misclassified fit banknotes into unfit ones, whereas type 2 errors indicate the opposite.

**Table 4:** Experimental results with testing data by Haar DWT and SVM classification. (*Denom*. and *Dir*. are denominations and directions, respectively. *Poly* indicates a polynomial kernel.) (unit: %).

Denom.	Dir.	SVM Kernel	Train 1—Test 2			Train 2—Test 1			Average EER
			Type 1 Error	Type 2 Error	EER	Type 1 Error	Type 2 Error	EER	
10 Rupee	A	linear	4.8889	0.0000	1.8841	0.0000	0.0000	0.0000	1.1764
	B	sigmoid	2.7273	0.0000	0.3448	0.0000	3.3333	0.5882	0.4575
	C	RBF	2.0000	0.0000	0.4762	0.0000	1.6667	0.0000	0.2779
	D	RBF	0.6667	3.3333	0.9524	0.0000	1.6667	0.0000	0.5555
20 Rupee	A	RBF	0.0000	0.0000	0.0000	1.2903	40.0000	2.2581	1.1290
	B	RBF	0.0000	5.0000	0.0000	0.0000	0.0000	0.0000	0.0000
	C	RBF	0.0000	0.0000	0.0000	0.0000	35.0000	1.3514	0.7142
	D	linear	0.0000	0.0000	0.0000	0.0000	0.0000	0.0000	0.0000
50 Rupee	A	RBF	0.4545	16.6667	2.8947	2.0833	0.0000	0.0000	2.2385
	B	linear	0.4545	0.0000	0.4545	3.7500	0.0000	0.0000	0.2275
	C	linear	0.4348	1.0000	0.3030	0.0000	0.0000	0.0000	0.1786
	D	RBF	0.4348	0.0000	0.3030	2.1739	0.0000	0.4348	0.3573
100 Rupee	A	linear	0.0000	12.0000	0.9524	0.0000	12.0000	0.1333	0.5605
	B	linear	0.0000	4.0000	0.0000	0.8333	7.5000	2.0968	1.3266
	C	linear	0.0000	0.0000	0.0000	0.0000	0.0000	0.0000	0.0000
	D	linear	0.2703	0.0000	0.0000	0.0000	23.3333	0.1316	0.0671
500 Rupee	A	poly	0.4651	0.0000	0.0000	0.0000	0.0000	0.0000	0.0000
	B	sigmoid	0.0000	50.0000	0.2381	1.1111	0.0000	0.0000	0.1190
	C	sigmoid	2.9545	0.0000	0.0000	0.0000	0.0000	0.0000	0.0000
	D	sigmoid	3.1111	0.0000	0.0000	0.0000	3.3333	0.0000	0.0000
Average EER									0.4693

**Table 5:** Experimental results with testing data by Daubechies DWT and SVM classification. (*Denom*. and *Dir*. are denominations and directions, respectively. *Poly* indicates a polynomial kernel.) (unit: %).

Denom.	Dir.	SVM Kernel	Train 1–Test 2			Train 2–Test 1			Average EER
			Type 1 Error	Type 2 Error	EER	Type 1 Error	Type 2 Error	EER	
10 Rupee	A	linear	4.2222	0.0000	0.3774	0.0000	1.6667	0.0000	0.2039
	B	linear	1.1364	0.0000	0.5882	0.0000	6.6667	0.1961	0.3944
	C	RBF	2.6667	0.0000	1.1765	0.0000	1.6667	0.3509	0.7405
	D	sigmoid	0.2222	8.3333	2.1569	0.6667	0.0000	0.0000	1.2499
20 Rupee	A	sigmoid	0.0000	0.0000	0.0000	1.2903	0.0000	1.2121	0.6249
	B	sigmoid	0.0000	0.0000	0.0000	0.0000	30.0000	0.5882	0.3126
	C	sigmoid	0.0000	0.0000	0.0000	0.3030	20.0000	0.3030	0.1515
	D	linear	0.0000	0.0000	0.0000	0.0000	0.0000	0.0000	0.0000
50 Rupee	A	linear	0.0000	0.0000	0.0000	0.8333	0.0000	0.0000	0.0000
	B	linear	0.4545	0.0000	0.0000	0.4167	0.0000	0.0000	0.0000
	C	linear	0.0000	3.0000	0.0000	3.0435	0.0000	0.0000	0.0000
	D	sigmoid	0.0000	0.0000	0.0000	1.3043	0.0000	0.4348	0.2175
100 Rupee	A	linear	0.0000	8.0000	0.5051	0.0000	8.0000	0.5556	0.5269
	B	RBF	0.4054	4.0000	1.2162	0.2778	5.0000	0.7500	0.9707
	C	linear	0.0000	0.0000	0.0000	0.0000	16.6667	0.0000	0.0000
	D	poly	0.4054	0.0000	0.3822	0.0000	26.6667	1.1650	0.8290
500 Rupee	A	linear	0.4651	0.0000	0.0000	0.0000	3.3333	0.0000	0.0000
	B	linear	0.0000	25.0000	0.0000	0.0000	0.0000	0.0000	0.0000
	C	linear	2.9545	0.0000	0.6522	0.0000	13.3333	0.0000	0.3334
	D	RBF	2.6667	0.0000	1.3115	0.0000	0.0000	0.0000	0.7548
Average EER									0.3655

**Table 6:** Cases of correct classification, type 1 errors, and type 2 errors in experiments on a 50-Rupee banknote (A-direction) using Haar DWT.

	Correct Classification		Type 1 Error Case	Type 2 Error Case
	Fit case	Unfit case		
Cropped ROI	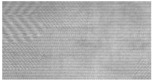	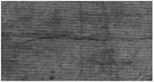	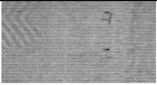	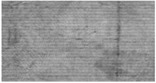
Image by Haar DWT	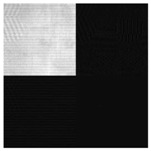	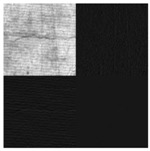	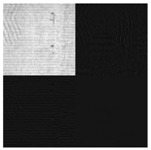	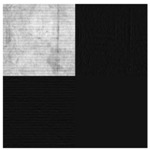

**Figure 6:**
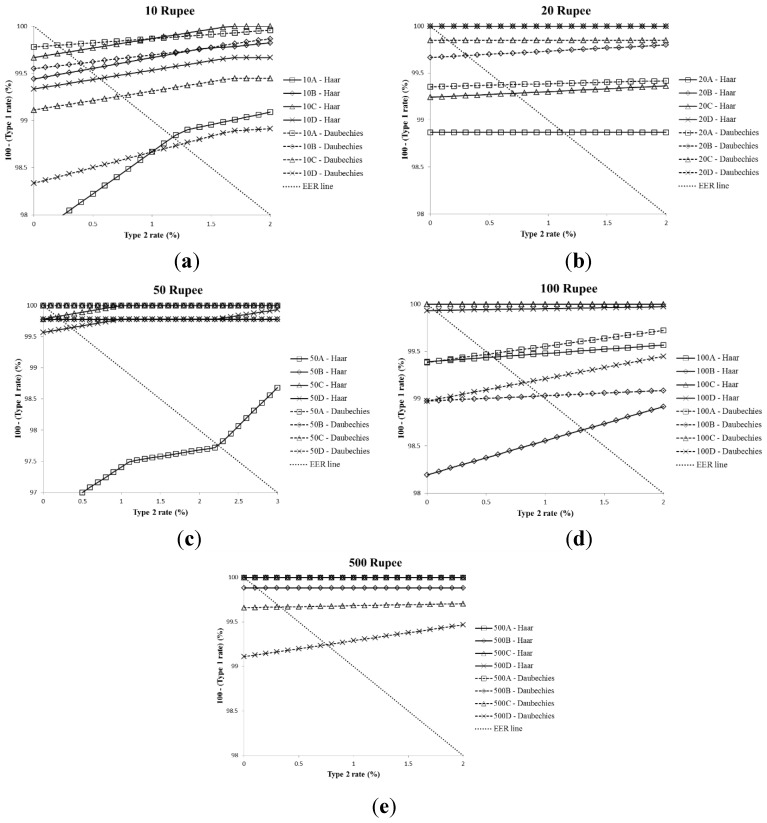
Average ROC curves of SVM testing process of the cases in Table 4 and Table 5: (**a**) 10 Rupee; (**b**) 20 Rupee; (**c**) 50 Rupee; (**d**) 100 Rupee and (**e**) 500 Rupee.

Figure 6 shows the average ROC curves of the testing datasets by two-fold cross-validation. From the results, we find that the average EER (0.3655%) by Daubechies DWT is slightly lower than the average EER (0.4693%) by Haar DWT. The lowest average EER was 0%, and the highest EER was 2.2385% with a 50-Rupee banknote (A-direction). The reason why the highest average EER was obtained with a 50-Rupee banknote (A-direction) is shown in Table 6.

Table 6 shows the examples of correctly classified and error cases in the classification results of a 50-Rupee banknote (A-direction) by Haar DWT. It can be seen from Table 6 that between the ROIs of fit and unfit banknote images, there are differences in gray levels: the unfit banknotes have darker images as well as higher pixel variance. This implies that unfit banknote images have a lower mean and higher standard deviation than fit banknote images. In the case of a type 1 error, the dirty marks on the fit banknote’s image result in a higher pixel variance, which can misclassify the banknote as unfit one. In the case of a type 2 error, the misclassified unfit banknote has bright pixels, which causes the misclassification of the unfit banknote into the fit one.

We also completed comparative experiments on the Indian banknote database using the method described in previous work [7]. Referring to [7], we extracted the features from the gray-level histogram of the banknote image in the interval of (161, 255) and used a multilayered perceptron (MLP) network as the classifier. The MLP network consists of 95 nodes in the input and hidden layers and one node in the output layer. For training process, we set the output values of the neural network to 1 in the cases of fit banknotes and to 0 in the cases of unfit ones. In these experiments, the MLP networks used a sigmoid kernel function, whose formula is given by Equation (4), and were trained by a back-propagation algorithm [24]:

(4) $$f(x)=\frac{1}{1+e^{-x}}$$

For training and testing, the same data in Table 4 and Table 5 were used for fair comparisons. We obtained the EERs from the MLP testing results by applying discriminant thresholds on the output of the MLP network. In this way, the banknotes that produced output values of MLP lower than the threshold were assigned as unfit notes, and those banknotes producing output values higher than the threshold were determined as fit notes. Table 7 compares the results of classification by our method with those of previous methods [7].

**Table 7:** Comparison of average EERs by our method with those by previous methods (unit: %).

Denomination	Direction	Haar DWT	Daubechies DWT	Previous Method [7]
10 Rupee	A	1.1764	0.2039	6.9036
	B	0.4575	0.3944	16.2962
	C	0.2779	0.7405	6.2792
	D	0.5555	1.2499	16.5487
20 Rupee	A	1.1290	0.6249	25.0000
	B	0.0000	0.3126	25.3456
	C	0.7142	0.1515	26.7717
	D	0.0000	0.0000	28.7490
50 Rupee	A	2.2385	0.0000	5.2397
	B	0.2275	0.0000	16.0191
	C	0.1786	0.0000	2.8302
	D	0.3573	0.2175	0.0000
100 Rupee	A	0.5605	0.5269	1.2179
	B	1.3266	0.9707	2.1053
	C	0.0000	0.0000	0.6868
	D	0.0671	0.8290	1.3765
500 Rupee	A	0.0000	0.0000	25.0000
	B	0.1190	0.0000	25.0000
	C	0.0000	0.3334	0.0000
	D	0.0000	0.7548	0.0000
Average EER		0.4693	**0.3655**	11.5685

As shown in Table 7, we can confirm that our method outperforms the methods used in previous works [7]. This is because of our feature extraction method, in which the features that best describe the fitness of Indian banknotes were selected by DWT and regression analysis. In addition, the range (161, 255) of gray-level values proposed in [7] is not optimal for all types of Rupee banknote images.

In general, the soiling on the banknote surface can independently occur on the front or back side of banknote. That is, the front side can be unsoiled whereas the back side can include the large amount of soiling, vice versa. Therefore, we discriminate the fit and unfit banknotes per each direction of A–D, separately. If we combine the results of multiple directions, the accuracy of discriminating the fit and unfit banknotes can be reduced. For example, if A direction is fit and C direction is unfit, the final result by combining these two information can be confused as fit or unfit. However, in actual banknote-counting machine or automatic teller machine (ATM), the input banknote should be determined as unfit one even if one of the four directions of A–D is determined as unfit. That is because this banknote can be jammed inside the machine if it is used, which increases the maintenance cost of dispatching the staff for repairing the machine. Therefore, we determine the fitness of banknote separately of A–D directions.

## 4. Conclusions

In this research, we proposed a new fitness classification method for Indian banknotes. The input banknotes were captured by visible light image sensors, and the ROIs were cropped from these banknote images. By using DWT and linear regression analysis, the discriminant features that had a high correlation to the reference data were extracted and fed into an SVM classifier for fitness determination. Experimental results showed highly accurate classifications of fit and unfit banknotes using denominations of 10, 20, 50, 100 and 500 Rupees.

In future works, we plan to do experiments using the proposed method on various types of banknotes such as US dollars, the Euro, and Korean banknotes. In addition, we would also consider extending the fitness levels to fit, medium, and unfit, instead of the binary classification of fit and unfit banknotes.

## References

[1] Geusebroek J.M., Markus P., Balke P. Learning Banknote Fitness for Sorting. Proceedings of the International Conference on Pattern Analysis and Intelligent Robotics.

[2] De Heij H. Durable Banknotes: An Overview. Presented at the BPC/Paper Committee to the BPC/General Meeting.

[3] Balke P. From Fit to Unfit: How Banknotes Become Soiled. Proceedings of the Fourth International Scientific and Practical Conference on Security Printing Watermark Conference.

[4] Buitelaar T. The Colour of Soil. Presented at the DNB Cash Seminar.

[5] Balke P., Geusebroek J.M., Markus P. BRAIN^2^—Machine Learning to Measure Banknote Fitness. Proceedings of the Optical Document Security Conference.

[6] Aoba M., Kikuchi T., Takefuji Y. (2003). Euro banknote recognition system using a three-layered perceptron and RBF networks. IPSJ Trans. Math. Model. Appl..

[7] He K., Peng S., Li S. A Classification Method for the Dirty Factor of Banknotes Based on Neural Network with Sine Basis Functions. Proceedings of the International Conference on Intelligent Computation Technology and Automation.

[8] Rahman M.M., Poon B., Amin M.A., Yan H. (2014). Recognizing Bangladeshi currency for visually impaired. Commun. Comput. Inform. Sci..

[9] Chetan B.V., Vijaya P.A. (2012). A robust side invariant technique of Indian paper currency recognition. Int. J. Eng. Res. Technol..

[10] Verma K., Singh B.K., Agarwal A. Indian Currency Recognition Based on Texture Analysis. Proceedings of the Nirma University International Conference on Engineering.

[11] Sharma B., Kaur A., Vipan D. (2012). Recognition of Indian paper currency based on LBP. Int. J. Comput. Appl..

[12] Pathrabe T., Karmore S. (2011). A novel approach of embedded system for Indian paper currency recognition. Int. J. Comput. Trends Technol..

[13] Sanjana M., Diwakar M., Sharma A. (2012). An automated recognition of fake or destroyed Indian currency notes in machine vision. Int. J. Comput. Sci. Manag. Stud..

[14] Choi E., Lee J., Yoon J. Feature Extraction for Bank Note Classification Using Wavelet Transform. Proceedings of the 18th International Conference on Pattern Recognition.

[15] Ahangaryan F.P., Mohammadpour T., Kianisarkaleh A. Persian Banknote Recognition Using Wavelet and Neural Network. Proceedings of the International Conference on Computer Science and Electronics Engineering.

[16] Gonzalez R.C., Woods R.E. (2010). Digital Image Processing.

[17] Nguyen D.T., Park Y.H., Shin K.Y., Kwon S.Y., Lee H.C., Park K.R. (2013). Fake finger-vein image detection based on fourier and wavelet transforms. Digit. Signal. Process..

[18] Simple Linear Regression. http://en.wikipedia.org/wiki/Simple_linear_regression.

[19] Coefficient of Determination. http://en.wikipedia.org/wiki/Coefficient_of_determination.

[20] Densitometer. http://www.xrite.com/documents/literature/gmb/en/100_d19_en.pdf.

[21] State Bank of India, Request for Proposal. http://www.sbi.co.in/portal/web/home/tenders-awarded.

[22] Hsu C.W., Chang C.C., Lin C.J. (2003). A Practical Guide to Support. Vector Classification.

[23] Chang C.-C., Lin C.-J. (2011). LIBSVM: A library for support vector machines. ACM Trans. Intell. Syst. Technol..

[24] Hagan M.T., Demuth H.B., Beale M.H. (1995). Neural Network Design.

